# Influence of perioperative dexamethasone on delayed union in mandibular fractures: A clinical and radiological study

**DOI:** 10.4317/medoral.20553

**Published:** 2015-08-04

**Authors:** Johanna Snäll, Satu Apajalahti, Anna-Liisa Suominen, Jyrki Törnwall, Hanna Thorén

**Affiliations:** 1 MD, DDS PhD. Department of Oral and Maxillofacial Diseases, University of Helsinki and Helsinki University Central Hospital, Helsinki, Finland; 2 DDS, PhD. Medical Imaging Centre, Helsinki University Central Hospital, Helsinki, Finland; 3 DDS, PhD, MSc. Institute of Dentistry, University of Eastern Finland, Kuopio, Finland. Department of Oral and Maxillofacial Surgery, Kuopio University Hospital, Kuopio, Finland. Department of Environmental Health, National Institute for Health and Welfare, Kuopio, Finland

## Abstract

**Background:**

The aim was to clarify the occurrence of delayed union after surgical treatment of mandibular fracture and investigate whether an association exists between perioperative use of dexamethasone and delayed union.

**Material and Methods:**

Thirty-seven patients were included in a prospective randomized study. Of these patients, 19 (51.4%) were randomized to receive a total dose of 30 mg of dexamethasone and 18 (48.6%) served as controls. Patients underwent clinical and radiological investigation immediately, one month, three months and six months postoperatively. Radiographs were evaluated by an experienced, blinded senior oral radiologist.

**Results:**

Delayed fracture union was found in 9 patients (24.3%). It was associated significantly with angle fractures (*p*=0.012). Delayed union occurred more frequently in patients who received dexamethasone (36.8%) than in those who did not (11.1%) (*p*=0.068). The association of infection with delayed union was significant (*p*=0.027). Moreover, dexamethasone was significantly (*p*=0.019) associated with delayed fracture union with concomitant infection. Gender, age group, smoking habit, treatment delay and duration of surgery were not associated with delayed union.

**Conclusions:**

Infection was associated with delayed union. Short-term high-dose dexamethasone predisposed to complicated fracture union, especially in patients with angle fractures. The relationship between dexamethasone and delayed bone healing without infection remains unresolved.

**Key words:**Mandibular, radiology, fracture union, dexamethasone.

## Introduction

Glucocorticoid therapy has well-known effects on bone density. These effects have been recognized after short-term application of glucocorticoids via several mechanisms. Short-term glucocorticoids have been shown to change osteocyte and osteoblast activity (1). They also suppress osteoclasts’ bone-degrading capacity (2) and increase bone resorption (1).

Previously, Li *et al*. presented a clinically interesting finding of short-term dexamethasone and delayed ossification in rats (3). A bone defect was created at the inferior border of the rat´s mandible. A dose of 0.4 mg/kg/24 hours was administered over 5 days, and postoperative ossification was evaluated radio logically and histologically. The authors observed that dexamethasone delayed the osteogenic differentiation and maturation of callus significantly from day 1 to day 10. However, the authors reported that delayed ossification later reverted to the level of the control group.

We have earlier clarified the association of high-dose perioperative dexamethasone with disturbances in surgical wound healing in mandibular fracture patients (4). Disturbances occurred slightly more in the dexamethasone group, but without statistical significance. After Li *et al*. published their findings (3), we reflected on the potential impact of dexamethasone on bone. To our knowledge, there are no previous clinical human studies on the influence of short-term glucocorticoids on ossification after mandibular fracture. Therefore, we decided to survey the bone repair in mandible fracture patients from a radiological view.

The aim of this study was to elucidate the occurrence of delayed union after surgical treatment of mandibular fracture and whether an association exists between perioperative use of dexamethasone and delayed union.

## Patient and Methods

- Study design

Patients included in the study were drawn from a larger cohort of healthy dentate patients aged 18 years or more who had participated in a randomised study aimed at clarifying the benefits of dexamethasone on pain, oedema and nausea after open reduction and fixation of a facial fracture. We excluded patients with infected fractures, a history of liver or kidney dysfunction, a history of peptic ulcer, a history of psychosis due to steroid use, pregnancy, breast feeding or allergy to any constituent of the dexamethasone preparation used.

For each facial fracture type, patients were randomly assigned to one of two groups. The patients in the study group received dexamethasone (Oradexon®), whereas patients in the control group received no glucocorticoid. Patients in the study group received a total dose of 30 mg of dexamethasone divided into three doses: 10 mg intravenously during anesthesia induction and two additional 10 mg doses intramuscularly every 8 hours over 16 hours. All patients received antibiotics until the seventh to tenth postoperative day, starting with three 1.5-g doses of cefuroxime taken intravenously at the ward during the first 24 hours post operatively and followed by three daily doses of 500 mg of cephalexin taken orally. Patients with allergy received four doses of clindamycin via corresponding routes.

The patients were scheduled for clinical follow-up at 1 day, 2 days, 1 week, 1 month, 3 months and 6 months after the operation. The clinical examination was conducted by a blinded investigator. In addition, patients were scheduled for panoramic imaging (Soredex Co., Tuusula, Finland) and Towne projection imaging (Samsung XGeo, Suwon, South Korea) preoperatively, immediately after surgery and at 1, 3 and 6 months after surgery. Patients with fractures in the anterior region of the mandible additionally underwent imaging with Scanora zonograms (Soredex Co., Tuusula, Finland).

- Inclusion criteria

Patients included in the present analysis had sustained one or two fractures in dentate areas of the mandible and had undergone open reduction and fixation with the aid of titanium mini plates. Fracture types included were the following: 1) a single fracture in the angle, 2) a single fracture in the body, 3) a single fracture in the symphysis/parasymphysis or 4) a double mandibular fracture (i.e., angle + body, angle + symphysis/parasymphysis fracture).

All fractures were fixated using an intraoral approach with the aid of 2.0-mm mini plates and non-locking mono cortical screws. Transbuccal approach was not used. Symphysis/parasymphysis fractures were fixated with two mini plates, and fractures in the mandibular body and angle were fixated with one mini plate according to the technique described by Champy and Lodde (1976) (5).

In order for a patient to be included in the analysis, a minimum radiological follow-up time of 3 months was required.

- Study variables

A senior oral radiologist (S.A.) evaluated the radiographs blindly twice with a 2week interval. The radiographs were analysed on a Dome E2 grayscale display (size 19.6″; Planar Systems, Beaverton, OR, USA) with a display resolution of 1200 x 1600 pixels. Since there was no disagreement between the reviews, intraobserver reliability was considered good/excellent.

The outcome variables were 1) delayed fracture union (including all patients with delayed fracture union), 2) delayed fracture union with associated infection (including patients with delayed fracture union who at any time during follow-up had an infection at the surgical site) and 3) delayed fracture union without associated infection (including patients who did not have any surgical site infection during follow-up).

The definition of radiographic union was based on a combination of the visibility of lucent fracture line, presence of callus and bridging of callus. In fractures involving the symphysis area, presence of callus visible at the inferior margin of the mandible was evaluated from a panoramic radiograph, whereas in angle fractures presence of callus visible on buccal cortical plate was evaluated from a Towne view. Delayed union was established when osteolysis and/or enhanced resorption was evident one month after surgery and/or the fracture line was still clearly visible three months after surgery without bridging of callus. In cases where normal resorption of the fracture line was evident one month post operatively, but fading of the fracture line and cortical continuity were observed three months post operatively, the radiographic union was established as successful.

The predictor variables were gender, age, smoking habit, fracture site, treatment delay, duration of surgery, perioperative use of dexamethasone and surgical site infection during follow-up.

- Statistical analysis

The significance of associations between radiologically defined delayed union (overall and also separately with or without associated infection) and the perioperative use of dexamethasone, surgical site infection, gender, age group, smoking habit, fracture site, treatment delay and duration of surgery were tested with Chi-square test. Differences between means of age (years), treatment delay (days), and duration of surgery (minutes) according to the occurrence of radiologically delayed union were tested with Wilcoxon two-sample test due to skewed distributions.

- Ethical approval

The Ethics Committee of the Department of Surgery and the Internal Review Board of the Division of Musculoskeletal Surgery, Helsinki University Central Hospital, Finland, approved the study protocol (Dno 33/E6/06). Informed written consent was obtained from all patients.

## Results

Of the patients recruited for the initial study, 49 fulfilled the inclusion criteria for the present analysis. Of these, 4 refused to participate. Of the remaining 45 patients, 8 were excluded: 4 because they failed to attend the required radiological investigations, one because he required additional surgery due to unsatisfactory fracture reduction, two because they failed to complete all scheduled medical doses, one because of an infection of a tooth situated in the fracture line. Thus, a total of 37 patients were followed up clinically and radiologically for at least 3 months.

Descriptive statistics of patients are shown in  Table 1. A total of 49 fracture lines were diagnosed in 37 patients, angle fractures (n=25) being most frequent. Nineteen patients received dexamethasone. Ten patients had surgical site infection during follow-up.

**Table 1:**
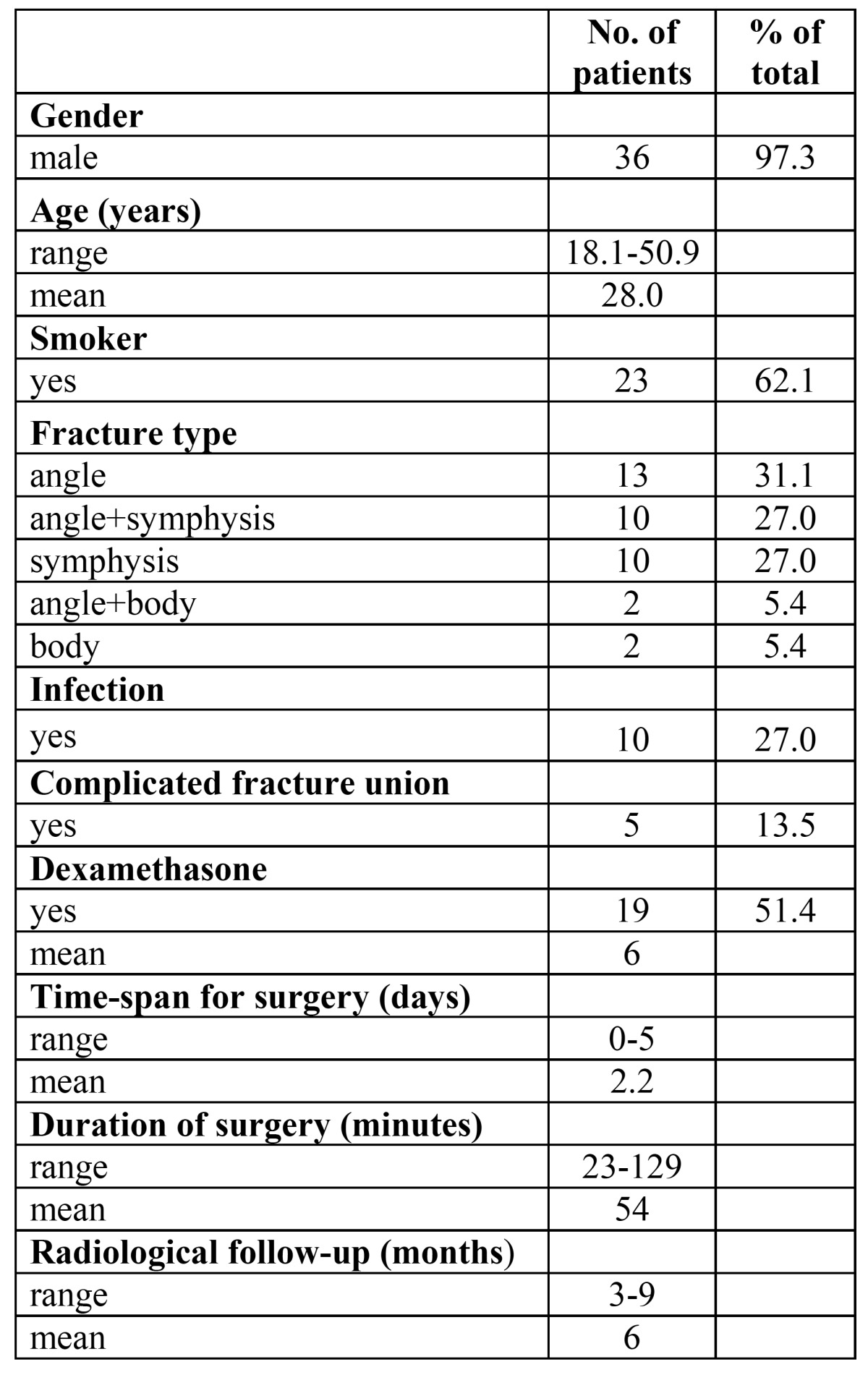
Descriptive statistics of 37 patients with mandibular fracture in the tooth-bearing area.

**Figure 1:**
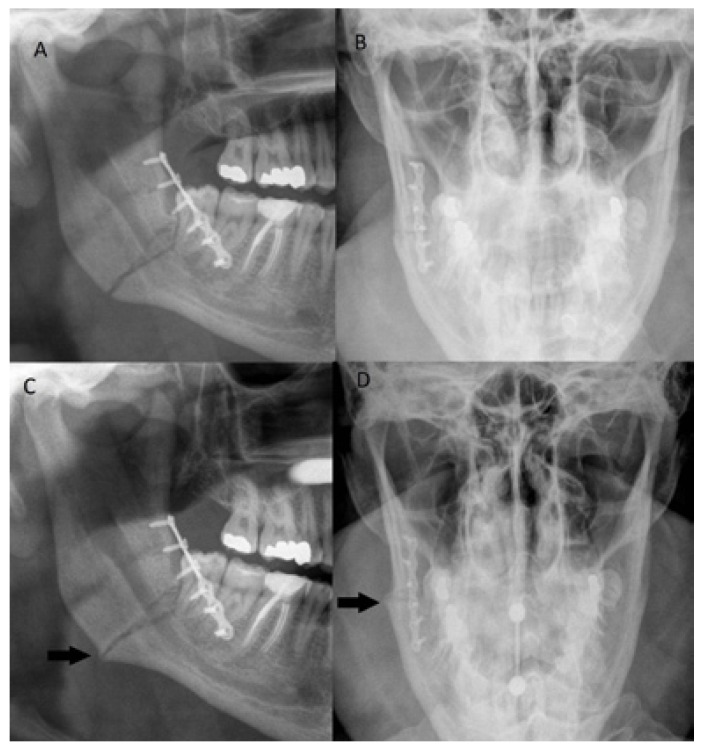
Panoramic and Towne view radiographs of right mandibular angle fracture with delayed fracture union taken immediately postoperatively (A, B) and three months postoperatively (C, D). In radiographs taken three months postoperatively fracture line is still clearly visible. Some callus formation is visible (arrows in C and D), however, no cortical continuity or callus bridging is present. The patient had a tongue piercing in C and D causing some artefact.

Delayed fracture union was observed in 9 patients (24.3%) (Fig. 1). None of the patients developed nonunion.  Table 2 shows the association between predictor variables and delayed fracture union. Surgical site infection (*p*=0.027) and angle fracture (*p*=0.012) were significant predictors for delayed union. Delayed fracture union occurred rarely in symphysis fractures (*p*=0.045). No association was present between delayed fracture union and gender, age group, smoking habit, treatment delay or duration of surgery.

**Table 2:**
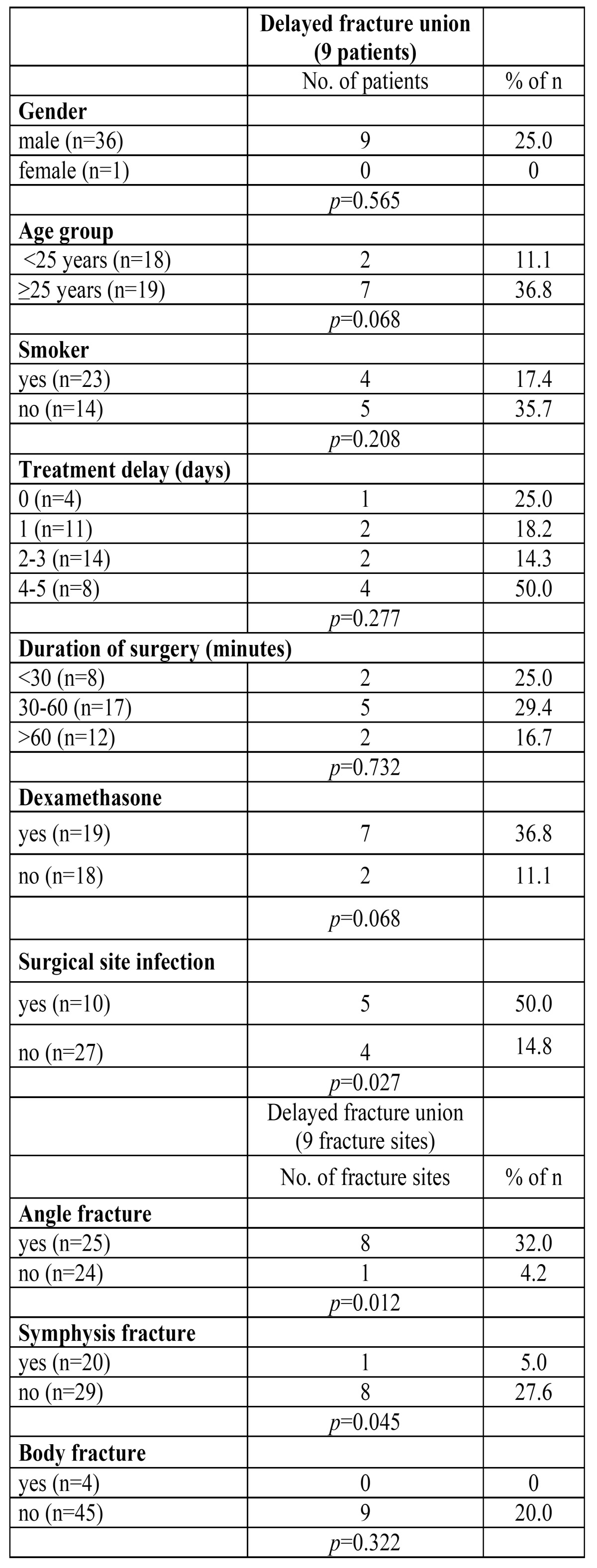
Association between predictor variables and delayed fracture union.

 Table 3 summarizes the association between predictor variables and delayed fracture union with surgical site infection and delayed fracture union not accompanied by infection. Dexamethasone was a significant predictor for delayed fracture union with associated infection (*p*=0.019). No other associations were found between examined predictors and delayed fracture union with or without infection.

**Table 3:**
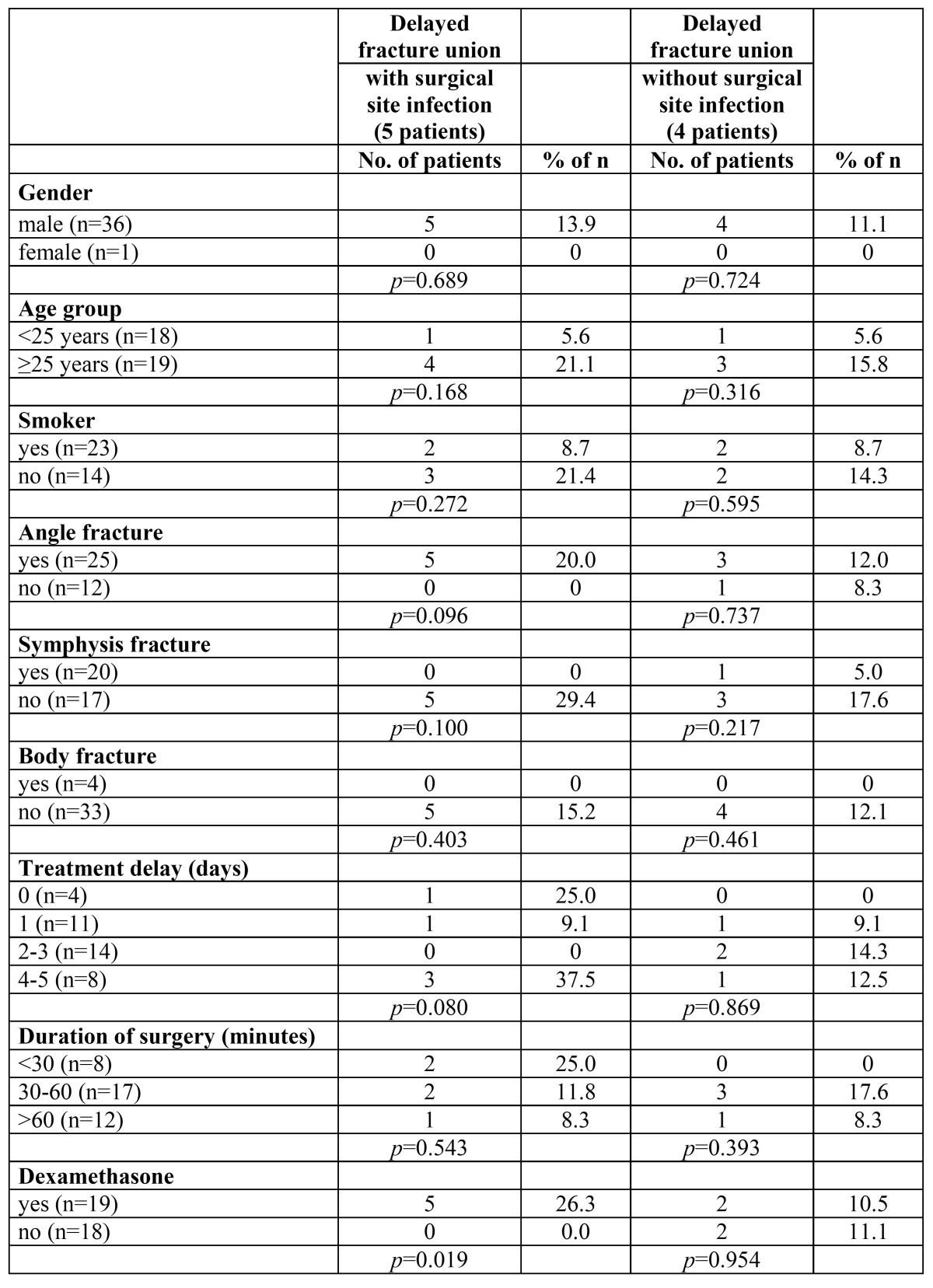
Association between predictor variables and delayed fracture union with and without surgical site infection.

## Discussion

We investigated the occurrence of delayed union after surgical treatment of mandibular fracture and whether an association exists between perioperative use of dexamethasone and delayed union.

Delayed union was observed in nine patients (24.3%). It was associated with surgical site infection (*p*=0.027) and angle fracture (*p*=0.012). Moreover, the association between dexamethasone use and delayed fracture union with infection was significant (*p*=0.019).

Infections were associated with delayed union. Also all five patients with delayed union associated with infection received dexamethasone. Consequently, none of the patients with infection who did not receive dexamethasone developed delayed union. Thus, dexamethasone seems to complicate fracture union by infection. In addition to effects on bone formation (1,2), glucocorticoids are known to inhibit both the growth of blood vessels (6,7) and angiogenesis (8). They are also well-known for their immunosuppressive and anti-inflammatory actions. Thus, glucocorticoids have several mechanisms by which they can predispose to delayed fracture union. Our study suggests that infections play a significant role in glucocorticoid-mediated ossification delay. However, the small sample size in the study is a shortcoming, and further studies are needed to verify the conclusion.

Previously, our study of mandibular fractures (4) revealed that disturbance in surgical wound healing was more frequent, albeit not significantly, among patients who had received dexamethasone. Moreover, our prospective study of perioperative dexamethasone in 64 patients with zygomatic complex fractures showed a significant association between disturbance in surgical wound healing and perioperative use of dexamethasone (9). We also found an association between clinical signs of surgical site infection and perioperative dexamethasone; in that study, all infections occurred in patients who received perioperative dexamethasone. The results of the present study suggest that high-dose perioperative dexamethasone should be considered a complicating factor of the surgical site also in mandibular fractures. Thus, dexamethasone seems to predispose to infectious complications with ossification delay in mandibular fracture patients. Therefore, perioperative high-dose dexamethasone cannot be recommended in mandibular fracture patients.

Delayed union was found in 9 patients, but only 5 of these patients had clinical symptoms at the fracture site and required additional treatment for infection associated with impaired bone repair. Thus, a radiologically observed delayed union is alone not a significant finding from the patient´s point of view. Radiological findings must be considered together with clinical status, requiring good collaboration between the radiologist and the surgeon.

The angle region was associated with delayed union in eight of nine patients. Moreover, in all five patients who had delayed union with infection, delayed union was located in the angle region. Fractures in the mandibular angle are known to be associated with more infectious complications (10,11). Ellis showed increased infection risk related to angle fractures when there were wisdom teeth present (12). Also large surface area of the fracture, impaired functions of the periosteum due to fracture and/or dissection, or diastasis of fracture ends might predispose angle fractures to delayed union compared with other parts of the dentate mandible. Careful radiological follow-up is recommended with regular clinical evaluation in patients with angular fractures, especially when potential factors for delayed bone healing are present.

In conclusion, perioperative high-dose dexamethasone predisposed patients to delayed fracture union with concomitant infection at the surgical site. However, the effect of dexamethasone on delayed fracture union without associated infection remains to be elucidated.
